# Radiological comparison of isolated syndesmotic screw fixation and transplate syndesmotic screw fixation in distal tibiofibular syndesmotic injuries

**DOI:** 10.3389/fsurg.2026.1845220

**Published:** 2026-05-21

**Authors:** Tianming Yu, Jichong Ying, Dichao Huang, Jingwei Zhang, Yunqiang Zhuang, Lin An

**Affiliations:** Department of Traumatic Orthopedics, Ningbo Clinical Research Center for Orthopaedics, Sports Medicine & Rehabilitation, Ningbo No.6 Hospital, Ningbo, China

**Keywords:** anatomy, computer tomography, syndesmotic injury, syndesmotic reduction, syndesmotic screw fixation, x-ray

## Abstract

**Purpose:**

The purpose of the study is to compare isolated syndesmotic screw fixation and transplate syndesmotic screw fixation in distal tibiofibular syndesmotic injuries.

**Methods:**

In this retrospective study, 37 patients diagnosed with pronation-external rotation ankle fractures requiring screw fixation were enrolled between July 2023 and December 2024. Nineteen patients underwent isolated syndesmotic screw fixation. Another group of 18 patients received transplate syndesmotic screw fixation. Follow-up was conducted for a minimum duration of 12 months. Radiographic outcomes were assessed using CT scans and x-rays. The CT evaluations included measurements of screw entry point and trajectory, anterior tibiofibular distance, posterior tibiofibular distance, anteroposterior fibular translation, and fibular rotation, conducted within 24 h post-operation and at one year after syndesmotic screw removal. x-ray assessments focused on tibiofibular overlap, tibiofibular clear space, and medial clear space, also evaluated at the same time nodes.

**Results:**

Preoperative and intraoperative data, including age, sex, time from injury to surgery, and mean operating time, were comparable between the two groups (*p* > 0.05). Significant differences were observed in the screw entry points and trajectories (*p* < 0.05) between the groups. Postoperative ankle x-rays showed no significant differences in syndesmotic reduction quality between the groups (*p* > 0.05). However, postoperative immediate CT scans indicated that the transplate syndesmotic screw fixation group experienced a higher incidence of posterior tibiofibular distance widening (*p* < 0.05). The 12-month postoperative ankle CT scans showed that in both groups, patients with mild ankle malreduction or overtightening of distal tibiofibular syndesmosis almost achieved spontaneous reduction after the removal of the distal tibiofibular syndesmotic screws, while patients with severe malreduction or syndesmotic widening still exhibited persistent distal tibiofibular syndesmotic malreduction.

**Conclusions:**

According to radiological comparisons, the fibular plate may interfere with screw placement and increase the likelihood of malalignment of the distal tibiofibular syndesmosis.

## Introduction

Ankle fractures are among the most frequently encountered intra-articular fractures, comprising approximately 3.9% of all fractures in adults, with a particularly high incidence in young and middle-aged individuals (1). Reports indicate that about 10% of ankle fractures and 25% of those necessitating surgical intervention involve concomitant injuries to the distal tibiofibular syndesmosis (2). The primary mechanism of injury for the distal tibiofibular syndesmosis is external rotation combined with ankle dorsiflexion. During these movements, the wider, anterolateral portion of the talar dome pushes the fibula which spreads away from the tibia until the syndesmosis is damaged, if the force is large enough (3). According to Lauge-Hansen classification, the pronation external rotation (PER) ankle fracture is one of the most common fractures that causes severe distal tibiofibular syndesmotic injury (4, 5). The fibular fracture line lies above the ankle joint, often high on the fibula (6). The distal tibiofibular syndesmosis is completely disrupted, permitting the talus to oscillate within an enlarged mortise. This results in talus instability and compromised ankle stability (7, 8).

For mild distal tibiofibular syndesmosis injuries, where there is no significant ankle instability, disruption of the tibiofibular ligaments, ankle fractures, or damage to the deltoid ligament, conservative treatment is generally advisable (9). This approach typically involves immobilization with a cast and avoiding weight-bearing on the affected limb. However, when there is significant instability of the distal tibiofibular joint or conservative treatment fails to maintain ankle stability, surgical intervention is necessary to restore the stability of the Neer circle of the ankle joint (10). The PER ankle fractures often require surgical treatment for distal tibiofibular syndesmotic injuries.

Research by Sagi et al. indicates that patients with malunited injuries of the distal tibiofibular syndesmosis experience significantly poorer postoperative functional recovery compared to those whose injuries are anatomically reduced (11). Similar studies have also identified that malalignment in the reduction of the distal tibiofibular syndesmosis is a key factor affecting functional recovery (12–14). Therefore, achieving an anatomical reduction and reconstruction of the distal tibiofibular syndesmosis to restore its stability and prevent the onset of post-traumatic arthritis in the ankle joint is a crucial aspect of treating ankle injuries.

When surgical intervention is required for patients, the available techniques include distal tibiofibular syndesmosis screw fixation, loop plate systems, suture anchor fixation, and reconstruction of the tibiofibular ligaments (15). Despite the numerous biomechanical, cadaveric and clinical studies concerning ankle fractures and syndesmotic injury, there is no common management consensus yet (16). However, the most commonly used method in clinical practice remains the distal tibiofibular syndesmosis screw fixation, which is also recommended by the AO Foundation (17).

In clinical practice, syndesmotic screw is typically required in PER ankle fractures. Syndesmotic screws are generally placed 2–3.5 cm proximal to the ankle joint line (18, 19). Thus, if no plate is used or the distal end of a plate sits more than 3.5 cm proximal to the joint line, a standalone syndesmotic screw will be inserted. For mid- to distal fibular fractures managed with plating, the syndesmotic screw is often placed through a hole in the plate. We often find that when using a fibular plate for distal tibiofibular syndesmosis screw fixation, there are more issues with poor alignment of the syndesmosis, uneven gaps, and separation, which can lead to further degeneration of the ankle joint, traumatic arthritis, and pain during functional activities, adversely affecting the patient's quality of life and ability to work.

Therefore, this retrospective study compares and analyzes the radiological features of PER ankle fractures treated with or without a fibular plate during syndesmotic screw fixation, trying to provide guidance regarding the following four points in clinical practice: (i) the accuracy of x-ray and CT evaluation of the distal tibiofibular syndesmosis and the differences between them; (ii) syndesmotic reduction quality of the two surgical fixations; (iii) the impact of using a fibular plate on the effectiveness of syndesmotic screw fixation; (iv) changes in the syndesmotic reduction before and after screw removal.

## Methods

### Inclusion and exclusion criteria

Inclusion criteria: (i) patients suffered ankle fractures (Lauge-Hansen classification: PER type) with concomitant injuries to the distal tibiofibular syndesmosis requiring operation; (ii) patients could tolerate surgery and consented to surgical treatment with distal tibiofibular syndesmosis screw fixation;

Exclusion criteria: (i) patients suffered previous surgery for either side of peri-ankle fracture that affected surgical protocol; (ii) patients were lost to follow-up within 12 months.

### Patient information

In this retrospective study, patients with distal tibiofibular syndesmotic injuries requiring surgery who underwent distal tibiofibular syndesmotic screw fixation with or without a fibular plate were included in the study at a tertiary medical center in China between July 2023 and December 2024. The study was approved by the medical ethics committee of our Hospital (NBLY-L-2026-007). Before surgery, each patient underwent routine examinations using standard radiography and bilateral CT with reconstruction. In total, 19 cases (15 men and 4 women) with isolated syndesmotic screw fixation completed the study and were included in the final outcome analysis. Furthermore, another 18 patients (12 men and 6 women) in the transplate syndesmotic screw fixation group were enrolled for comparison. All fractures were treated by one surgeon who had more than 15 years of experience in peri-ankle fracture surgery. Surgical implants were provided by a single-device manufacturer (Wego Group Co., Ltd., China). Postoperative assessments were performed by two surgical fellows. Patient demographics are provided in detail in Table 1.

**Table 1:** Patient demographics.

Variable	Isolated Syndesmotic Screw Fixation	Transplate Syndesmotic Screw Fixation	*P* value
Patient volume	19	18	–
Mean age	46.63 ± 18.09	47.33 ± 12.51	0.892
Male (%)	78.95%	66.67%	0.476
Mean operating time (min)	88.68 ± 27.58	102.5 ± 27.67	0.137
Time form injury to surgery (day)	6.42 ± 6.67	6.06 ± 2.21	0.654

### Surgical procedure and postoperative rehabilitation

After subarachnoid block anesthesia, the patient's operative position (supine or lateral decubitus on the contralateral side) was determined intraoperatively based on the need for fixation of a posterior malleolar fracture. The operative limb had the tourniquet inflated. The maximal single duration of tourniquet was 90 min, and tourniquet would be released before the final closure. Upon completion of ankle fracture fixation, the determination of a syndesmotic injury was based on standard criteria using ankle stress radiographs showing widening of the tibiofibular clear space using an intraoperative dynamic Cotton or external rotation stress test (20). Fluoroscopic measurements indicative of syndesmotic diastasis included a tibiofibular clear space (TCS) of ≥5 mm, tibiofibular overlap (TFO) of ≤10 mm, or medial clear space (MCS) ≥4 mm on the anteroposterior (AP) view (21). If syndesmotic stabilization was indicated, clamp reduction of the syndesmosis was achieved by placing the lateral clamp tine on the fibular tubercle and the medial clamp tine on the anterior one-third of distal tibia within the same axial plane. A trans-syndesmotic screw was then placed approximately 2.5 cm proximal to the ankle joint, either through the existing plate or independently. In cases where a plate was present at the intended insertion site, the screw was introduced through a plate hole; otherwise, it was placed directly across the syndesmosis to achieve fixation. Fluoroscopic imaging was utilized to assess the ankle joint alignment, the distal tibiofibular syndesmosis spacing, and the position of the internal fixation hardware. The ankle was then immobilized in dorsiflexion using a plaster cast.

Patients would be splinted for 2 weeks until the wound healed. Patients were discharged from hospital when their vital signs were stable and there was no exudation from the incision. Under the supervision of a physiotherapist, non-weight-bearing functional rehabilitation was initiated, and gradual weight-bearing was allowed only after screw removal at 8–10 weeks postoperatively.

### Evaluation of syndesmotic reduction

At the postoperative follow-up, all patients were assessed with X-rays and CT scan of within 24 h. All fractures were reviewed with postoperative X-rays by an orthopedic surgeon during each clinic visit. One year after the removal of the syndesmotic screw, the patients would be required for CT scanning of the affected ankle.

**Figure 1:**
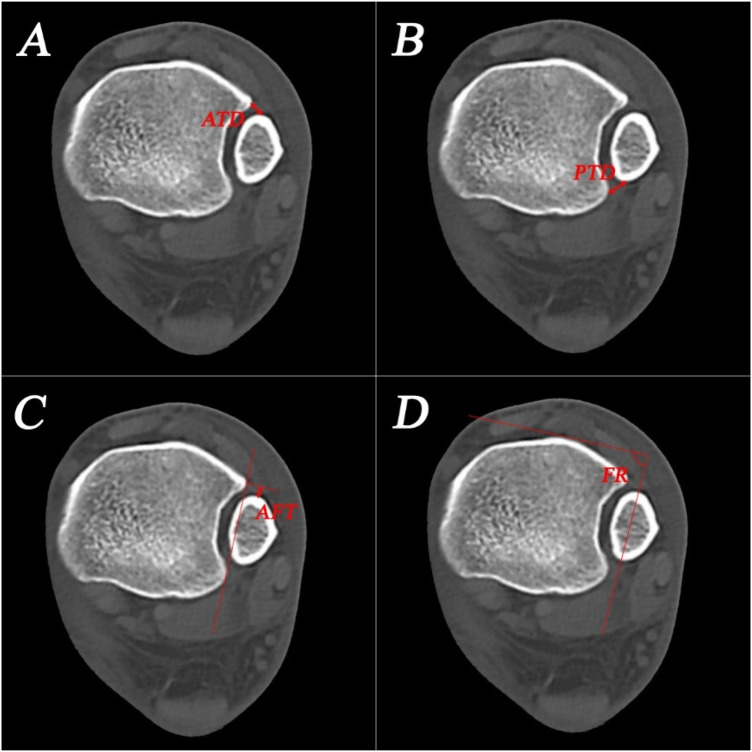
Measurements for evaluating syndesmotic reduction on CT images. **(A)** anterior tibiofibular distance (ATD); **(B)** posterior tibiofibular distance (PTD); **(C)** anteroposterior fibular translation (AFT); **(D)** fibular rotation (FR).

CT images were obtained with a slice thickness of 1 mm or less. The images were reformatted to produce a standardized axial image for analysis for each ankle, parallel to the long axis of the tibia (22). Four different measurements would be evaluated for tibiofibular reduction which have been reported to be relatively reproducible (23). The measurements included anterior tibiofibular distance (ATD, the distance between the most anterior point of the incisura and the most anterior point of the fibula. The difference between the injured and contralateral sides was used for analysis), posterior tibiofibular distance (PTD, the distance between the most posterior point of the incisura and the most posterior point of the fibula), anteroposterior fibular translation (AFT, a line is drawn perpendicular to the line connecting the anterior and posterior tibial tubercles, passing through the anterior tibial tubercle. The distance between this line and the most anterior point of the fibula is then measured) and fibular rotation (FR, angle between the tangent to the anterior tibial surface at its most anterior point and the bisection of the mediolateral midline of the fibula) (Figure 1). We considered an ankle to exhibit malreduction (namely positive value) if ATD, PTD, or AFT in the injured ankle was 2 mm or greater than that in the contralateral ankle, or if the difference in FR was 5°or greater (21).

**Figure 2:**
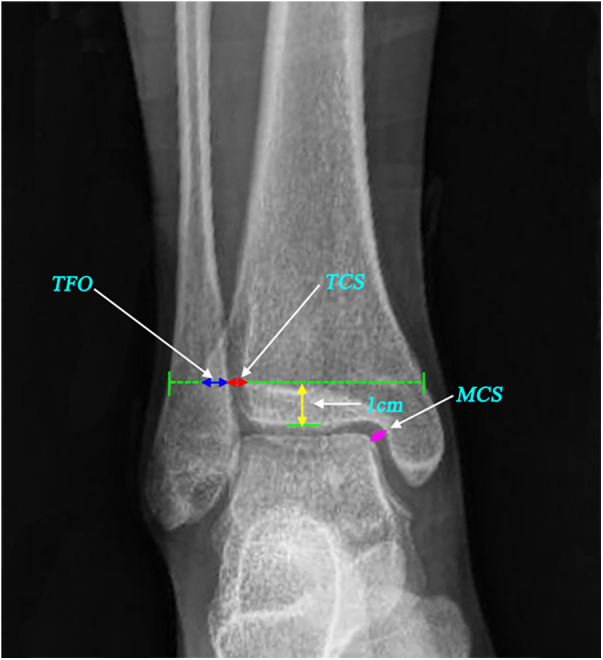
Measurements for evaluating syndesmotic reduction in the radiographs. Blue double-headed arrow: tibiofibular overlap (TFO); red double-headed arrow: tibiofibular clear space (TCS); pink double-headed arrow: medial clear space (MCS).

For each standard ankle AP radiograph, TFO (the distance between the lateral border of the anterior tibial prominence and the medial fibula 1 cm proximal to the tibial plafond), TCS (the distance between the lateral border of the posterior tibial malleolus and the medial aspect of the fibula measured 1 cm proximal to the tibial plafond), and MCS (the distance from the lateral border of the medial malleolus to the medial border of the talus at the level of the talar dome) were measured (Figure 2). The following criteria have been used to diagnose syndesmosis disruption on radiographs: TFO ≤ 10 mm, TCS ≥ 5 mm, and MCS ≥ 4 mm. The reduction quality of the ankle fracture was evaluated by two surgical fellows.

### Statistics

Descriptive statistics were recorded and collated. The mean and standard deviation for the patient demographics and intraoperative outcomes were calculated from the original dataset. An unpaired *t*-test was used to compare the patient demographics and intraoperative outcomes between the two groups. Fisher's exact test was used to measure the association between the categorical data of the two groups. Results were considered significant when *p* < 0.05. In addition, the study utilized kappa test for the consistence of 2 independent observers in evaluating the CT scan/X-ray and characterized kappa over 0.75 as excellent, 0.40–0.75 as fair to good, and below 0.40 as poor. All statistical analyses were performed using SPSS 22.0 (SPSS Inc., IL, USA).

## Results

### General results

Preoperative demographic characteristics did not differ significantly between the groups (*p* > 0.05; Table 1). The period between injury to surgery was 6.42 ± 6.67 days (range, 3–11 days) and 6.06 ± 2.21 days (range, 4–10 days) in the isolated syndesmotic screw fixation and the transplate syndesmotic screw fixation groups, respectively, with no significant difference between the groups (*p* = 0.654). The operation times also did not differ significantly between the isolated syndesmotic screw fixation (88.68 ± 27.58 min, range, 50–140 min) and the transplate syndesmotic screw fixation groups (102.5 ± 27.67 min, range, 50–150 min) (*p* = 0.137).

### Screw position

In evaluating the screw entry points and trajectories by kappa analysis, the coefficients of consistence reach 0.782 and 0.838 respectively. It proves that the evaluation results are highly credible. In the immediate postoperative CT evaluation, there were significant differences in both the screw entry points (*p* = 0.001) and the screw trajectories (*p* = 0.006) between the two groups. In the isolated syndesmotic screw fixation group, screw entry points were primarily located on the crest of the lateral malleolus, while in the transplate syndesmotic screw fixation group, entry points were mainly on the anterolateral surface. The trajectories of the screws in the isolated syndesmotic screw fixation group were generally parallel to the central axis of the distal tibiofibular syndesmosis. In contrast, trajectories in the transplate syndesmotic screw fixation group were more varied, with screws more frequently placed backward (Table 2).

**Table 2:** Distribution of screw positions on CT images.

Variable		Isolated Syndesmotic Screw Fixation	Transplate Syndesmotic Screw Fixation	*p* value
		Positive	Positive	
Screw entry point	Lateral malleolus crest	13	3	0.001
	Anterolateral surface	2	12	
	Posterolateral surface	4	3	
Screw trajectory	Parallel	18	9	0.006
	Forward	1	2	
	Backward	0	7	

### Radiological evaluation of distal tibiofibular syndesmosis

In evaluating MCS, TFO, TCS, ATD, PTD, AFT, and FR by kappa analysis, all parameters demonstrate good-to-excellent reliability (the coefficients of consistence reach from 0.706 to 1.000). Postoperative ankle X-rays showed no significant differences in syndesmotic reduction quality between the groups (*p* > 0.05). In the isolated syndesmotic screw fixation group, only one case (1/19) exhibited an increase in MCS, whereas in the transplate syndesmotic syndesmotic screw fixation group, two cases (2/18) showed an increase in the MCS. No abnormalities were found in TFO, TCS in either group.

Postoperative CT evaluation within 24 h revealed that in the isolated syndesmotic screw fixation group, 6 out of 19 patients (6/19) had malreduction of the distal tibiofibular syndesmosis, and in the transplate syndesmotic screw fixation group, 7 out of 18 patients (7/18) presented with malreduction, with no significant difference between the two groups (*p* = 0.737). However, further statistical analysis using ATD, PTD, AFT, and FR revealed a significant difference in PTD between the two groups (*p* = 0.044), with more patients in the transplate syndesmotic screw fixation group showing PTD widening.

The insertion of distal tibiofibular syndesmotic screw tends to be overtightening in the distal tibiofibular syndesmosis. 12 months postoperative CT scan showed that overtightening of the distal tibiofibular syndesmosis could achieve a certain degree of reduction after the removal of the distal tibiofibular syndesmotic screws, whereas tibiofibular syndesmotic widening couldn't be autonomously corrected (Tables 3, 4).

**Table 3:** Radiographic assessment of distal tibiofibular syndesmosis positioning.

Variable		Isolated Syndesmotic Screw Fixation		Transplate Syndesmotic Screw Fixation		*p* value
		Positive	Negetive	Positive	Negetive	
CT evaluation (24 h)	Patient	6	13	7	11	0.737
	ATD	2	17	2	16	1
	PTD	2	17	7	11	0.044
	AFT	2	17	2	16	1
	FR	4	15	7	11	0.295
CT evaluation (12 months)	Patient	5	14	7	11	0.495
	ATD	2	17	2	16	1
	PTD	2	17	7	11	0.044
	AFT	1	18	2	16	0.604
	FR	4	15	7	11	0.295
Anteroposterior X-ray (24 h)	Patient	1	18	2	16	0.604
	TFO	0	19	0	18	11
	TCS	0	19	0	18	1
	MCS	1	18	2	16	0.604
Anteroposterior X-ray (12 months)	Patient	1	18	2	16	0.604
	TFO	0	19	0	18	1
	TCS	0	19	0	18	1
	MCS	1	18	2	16	0.604

**Table 4:** Distribution of ATD or PTD counts across different time nodes.

Variable	Isolated Syndesmotic Screw Fixation			Transplate Syndesmotic Screw Fixation			*P* value
	Overtightening	Widening	Normal	Overtightening	Widening	Normal	
ATD or PTD (24 h)	13	5	20	15	9	12	0.199
ATD or PTD (12 months)	6	5	27	7	9	20	0.331

## Discussion

The study assessed two screw fixation types for the treatment of distal tibiofibular syndesmotic injuries in PER ankle fractures. According to results, the perioperative data, such as time form injury to surgery, mean operating time, were similar between two groups. However, the radiological outcomes, evaluated by x-rays and CT scans, showed some differences between two groups. Patients who underwent isolated syndesmotic screw fixation had better screw trajectories and less syndesmotic malreductions than those who underwent the transplate syndesmotic screw fixation.

### The evaluation accuracies of X-ray and CT scan

In the study, immediate postoperative anteroposterior and lateral ankle X-rays showed that in the isolated syndesmotic screw fixation group, one patient exhibited widening of MCS, and in the transplate syndesmotic screw fixation group, two patients showed MCS widening, while TFO and TCS were normal. CT scans confirmed that patients with MCS widening had malreduction at the distal tibiofibular syndesmosis. However, in patients who were assessed as normal on X-rays, CT scans still revealed some cases of malreduction at the distal tibiofibular syndesmosis. The X-ray cannot effectively assess the translation or rotational displacement of the distal fibula. Therefore, anteroposterior and lateral ankle X-rays have a false-negative rate in assessing the accuracy of distal tibiofibular syndesmotic reduction, and ankle CT scans with reconstruction are an effective means to assess the accuracy of reduction at the distal tibiofibular joint. This is also consistent with some previous research findings.(15, 24).

### The impact of the plate on screw placement

Starting approximately 2 cm above the ankle, the distal fibula's lateral surface often forms a triangular area, which we refer to as the lateral malleolar crest. Lateral malleolar crest is an important reference point for application of syndesmotic screws and a plate, as well as for assessment of the position of the distal fibula in the fibular notch (25). Based on preliminary imaging research, we found that the syndesmosis screw should ideally be inserted from the crest of the distal fibular triangular area to pass through the central axis of the syndesmosis. This ensures uniform compression of the syndesmosis during tightening.

**Figure 3:**
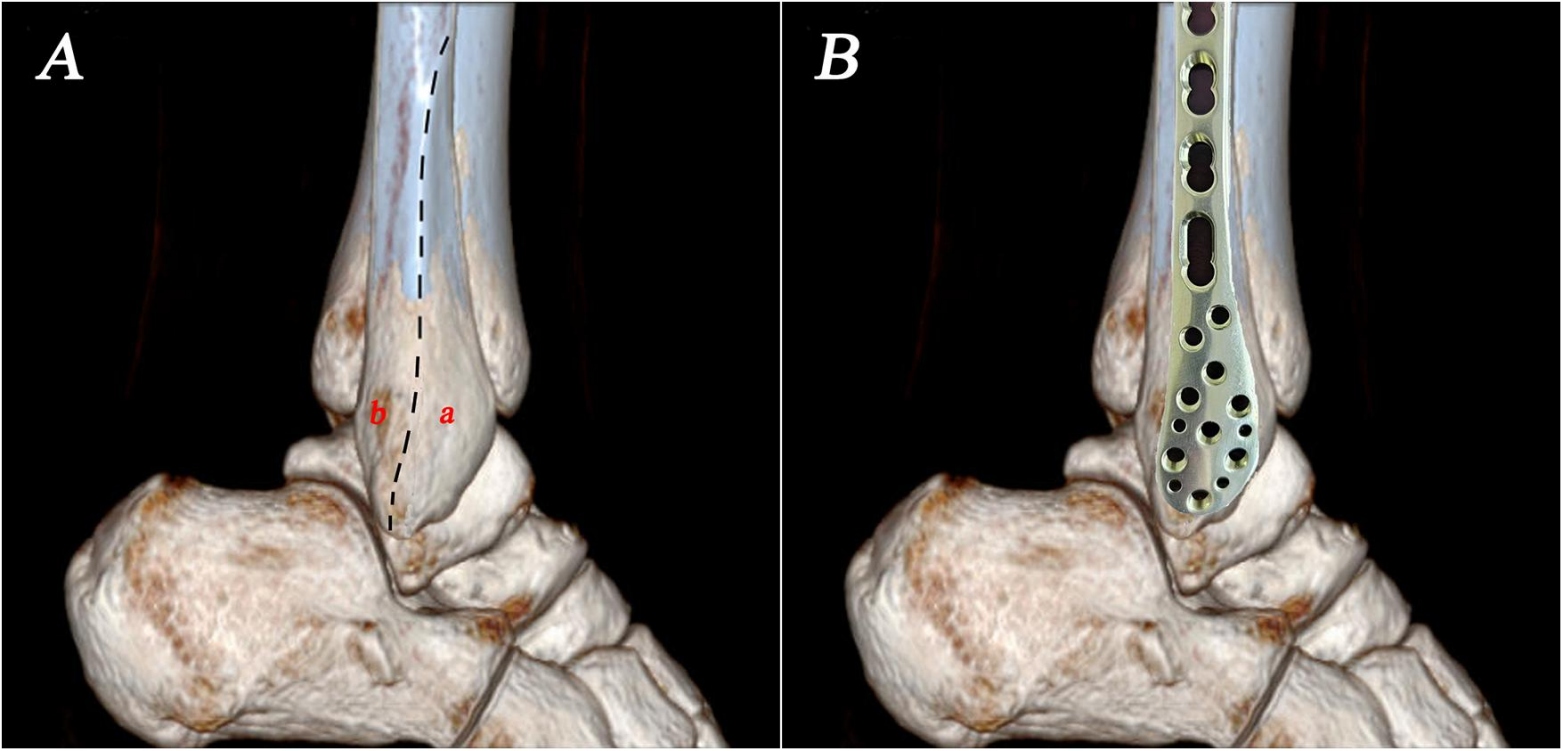
**(A)** Bony anatomy of the lateral surface of the distal fibula. Black dashed line: lateral malleolar crest; a: anterolateral aera of the distal fibula; b: posterolateral area of the distal fibula. **(B)** Conventional placement of a fibular plate on the lateral malleolar surface.

**Figure 4:**
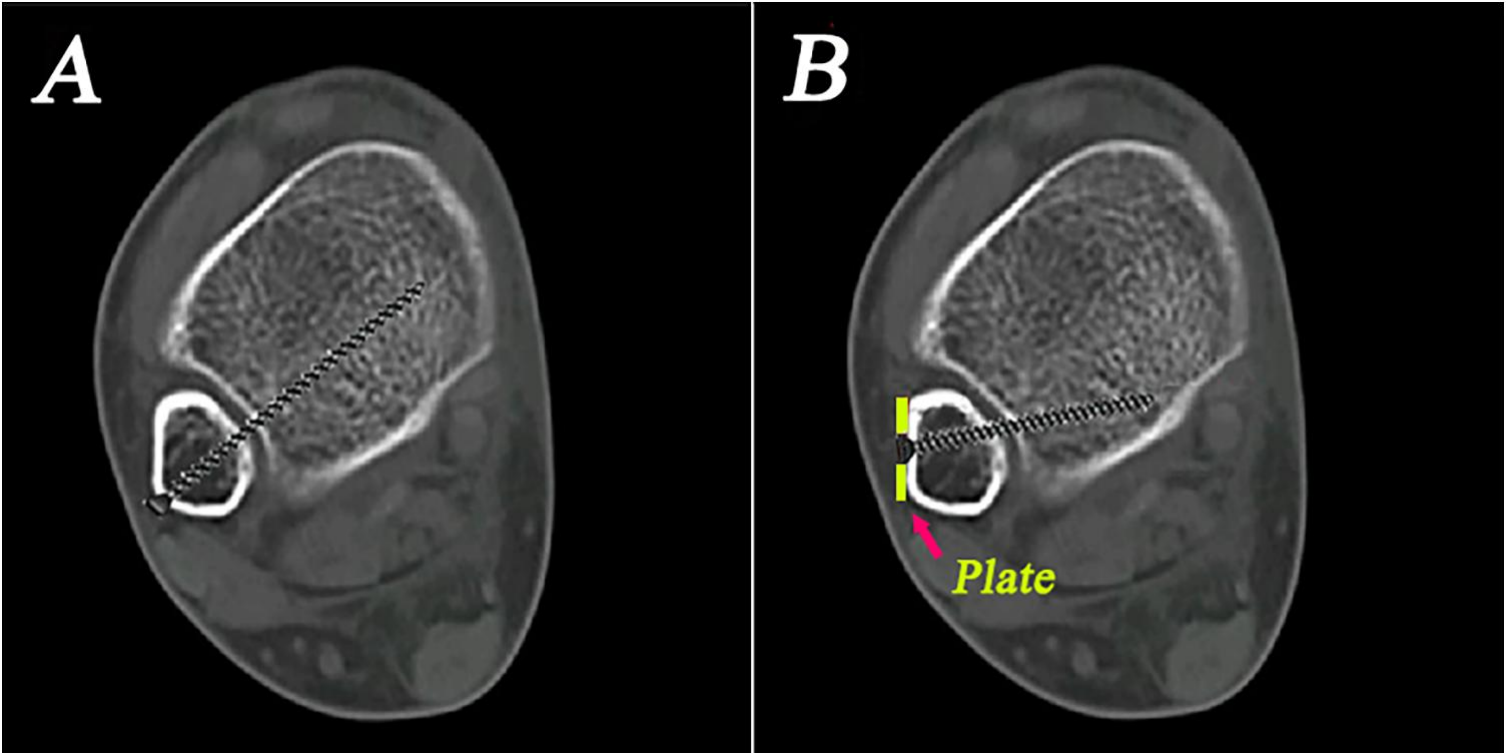
**(A)** Isolated syndesmotic screw fixation has better screw entry and trajectory. **(B)** The transplate syndesmotic screw entry points are more often located on the anterolateral surface of the fibula and screw trajectories are more frequently placed backward due to the interference of the plate.

**Figure 5:**
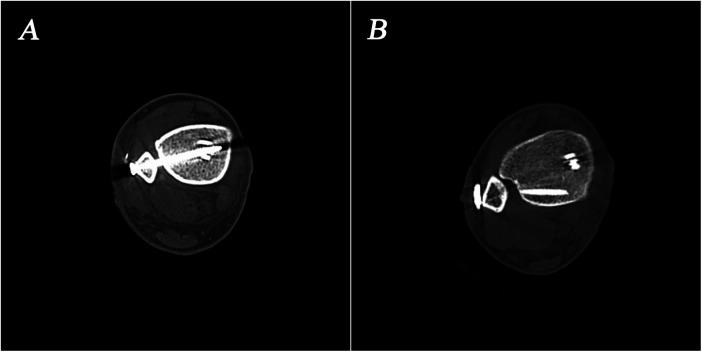
**(A)** Ideal placement of the syndesmotic screw for the treatment of distal tibiofibular syndesmotic injuries. **(B)** Due to plate interference, unplanned trajectory of syndesmotic screw insertion occurs, causing fibular rotation, overtightening of anterior tibiofibular distance, and widening of posterior tibiofibular distance, ultimately resulting in malalignment of the syndesmosis.

Ideally, the plate should respect the midline of the lateral aspect of the distal fibula. However, it usually covers the anterolateral surface of the triangular area to ensure optimal fit to the distal fibular bone surface (Figure 3). When the plate is positioned anteriorly, the standard trajectory of the syndesmosis screw inserted through the plate will inevitably be affected. As a result, the screw entry points were more often located on the anterolateral surface of the fibula and screw trajectories were more frequently placed backward in the transplate syndesmotic screw fixation group (Figure 4). This unplanned screw insertion may lead to inappropriate compression, causing fibular rotation, overtightening of ATD, and widening of PTD, ultimately resulting in malalignment of the syndesmosis. In addition, with the unplanned trajectory, an excessively long screw may protrude posteromedially, which can be easily overlooked on intraoperative imaging, risking injury to important structures (25) (Figure 5).

### The occurrence of syndesmotic malredcution and changes after screw removal

Although there was no significant difference in the incidence of malreduction in the distal tibiofibular syndesmosis between the two groups, we found a significant increase in patients with PTD widening in the transplate syndesmotic screw fixation group. In these patients, there is often an anterior entry and backward trajectory of the distal tibiofibular syndesmotic screw. The positioning of the screw often leads to uneven pressure on the distal tibiofibular syndesmosis, resulting in overtightening of the ATD and widening of the PTD after rotation of the fibula.

**Figure 6:**
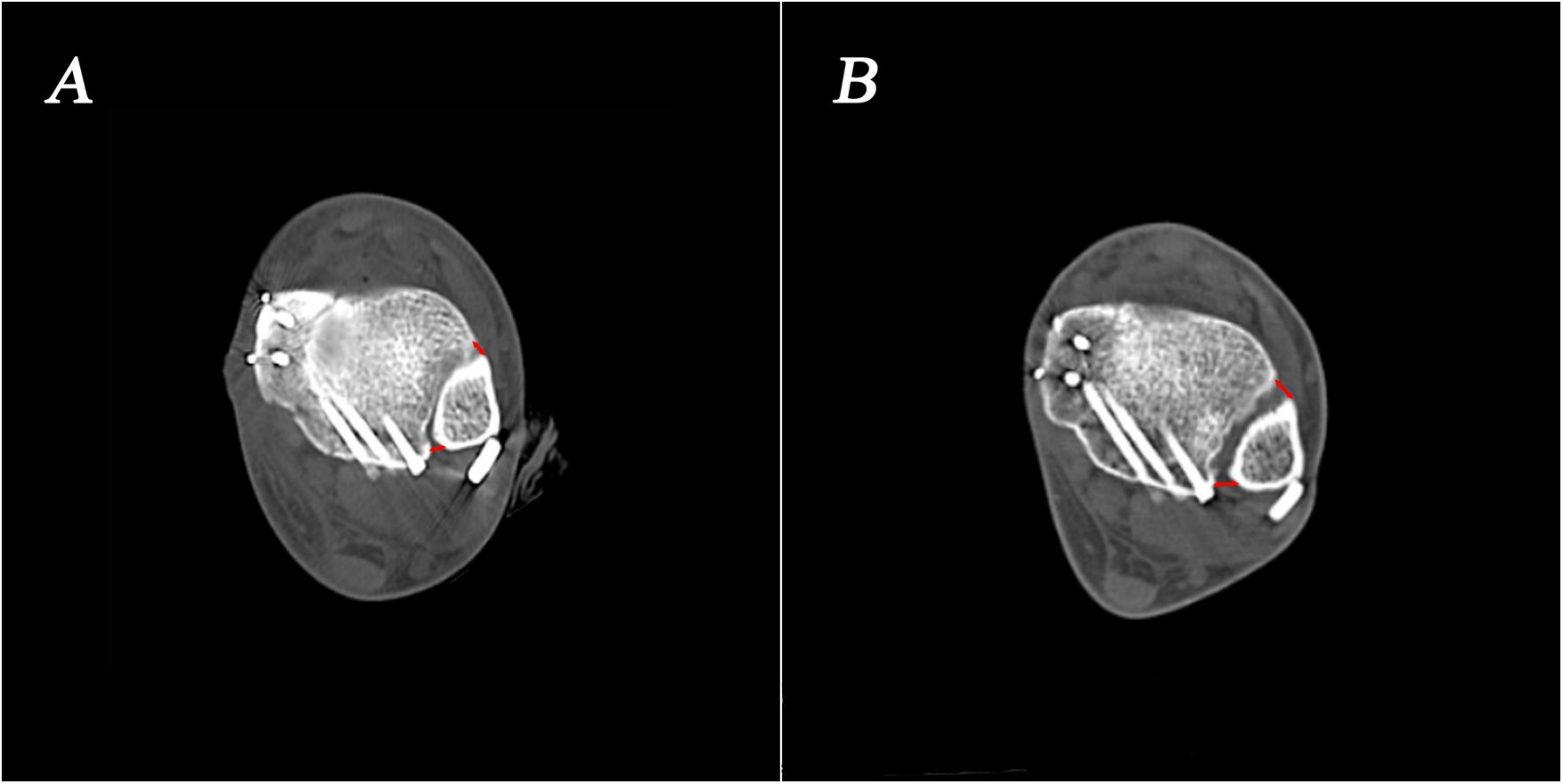
**(A)** The insertion of distal tibiofibular syndesmotic screw may be overtightening in the distal tibiofibular syndesmosis. **(B)** Overtightening of the distal tibiofibular syndesmosis could achieve a certain degree of reduction after the removal of the distal tibiofibular syndesmotic screws.

12 months after the removal of the distal tibiofibular syndesmotic screw, upon re-evaluation of the reduction of the distal tibiofibular syndesmosis, the study found that overtightening could achieve reduction, resulting in a well-restored distal tibiofibular syndesmotic space (Figure 6). However, in patients with ATD or PTD widening, the distal tibiofibular syndesmotic space did not achieve reduction and was wider than that before the screw removal.

### Strengths and limitation

To our knowledge, few studies have directly quantified the radiological impact of fibular plates on syndesmotic screw trajectory and reduction accuracy. The retrospective study included 37 cases of PER ankle fractures with reliable and complete data. As a result, the study identifies the influence and characteristics of fibular plates on the entry points and trajectories of screws during fixation of the distal tibiofibular syndesmosis, which may provide some guidance in clinical practice.

This study has several limitations. This retrospective study included 37 cases of PER ankle fractures, requiring a larger sample size, long-term follow-up and prospective multi-center research to enhance the reliability of the conclusions. Furthermore, as this is a radiological comparative study, the differences in ankle function between the two groups were not examined, highlighting the necessity for further research to address this gap. The challenge of mitigating the impact of the fibular plate on screw placement remains unresolved. Therefore, the development of a novel distal fibular plate specifically designed to accommodate distal tibiofibular syndesmotic screws may be required in clinical practice.

## Conclusion

The study compared the radiological differences between the isolated syndesmotic screw fixation and the transplate syndesmotic screw fixation. CT scans provide a more accurate assessment of the distal tibiofibular syndesmosis reduction. In the isolated syndesmotic screw fixation group, the entry points and trajectories of the syndesmotic screws were more standardized, suggesting that the fibular plate interferes with screw placement. The transplate syndesmotic screw fixation group exhibited a higher incidence of PTD widening. An overtightened syndesmosis achieved spontaneous reduction after the removal of the syndesmotic screw, whereas in patients with a widened syndesmosis, the increased gap persisted or even enlarged after screw removal, indicating that the interference from the fibular plate may increase the likelihood of persistent malalignment of the distal tibiofibular syndesmosis.

## Data Availability

The original contributions presented in the study are included in the article/Supplementary Material, further inquiries can be directed to the corresponding author.
